# Yeast Cell Cake Characterization in Alcohol Solution for Efficient Microfiltration

**DOI:** 10.3390/membranes11020089

**Published:** 2021-01-27

**Authors:** Nobuyuki Katagiri, Keisuke Tomimatsu, Keiichi Date, Eiji Iritani

**Affiliations:** 1 Department of Environmental Technology, Meijo University, 1-501 Shiogamaguchi, Tempaku-ku, Nagoya 468-8502, Japan; 2 Department of Chemical Engineering, Nagoya University, Furo-cho, Chikusa-ku, Nagoya 464-8603, Japan; tifamgreenline@gmail.com (K.T.); keiichidate@gmail.com (K.D.); iritani@nuce.nagoya-u.ac.jp (E.I.)

**Keywords:** microfiltration, yeast, alcohol fermentation, fouling, cake resistance, cake porosity

## Abstract

Microfiltration is widely used to remove microbial cells from the fermentation broth in the downstream processing of biotechnological products. Because filtration behaviors are strongly affected by the characteristics of the microbial cell cake formed on the surface of the membrane, insights into the cake structure facilitate the design and operation of filter equipment and membranes. In the alcohol fermentation process using a yeast strain, the cake characteristics are considered to be complicated because yeast cells are strongly influenced by external factors such as filtration pressure and alcohol concentration. In this study, we evaluated the membrane filtration properties, in particular the cake characteristics of a yeast suspension containing alcohol. Microfiltration experiments were performed in the dead-end filtration mode using yeast suspensions with several ethanol concentrations (0–20 wt%) under constant pressure. Flux decline behaviors caused by yeast cake were put in a similar form for 0–15 wt% ethanol concentrations. In contrast, a severe flux decline was observed for the suspension with 20 wt% ethanol concentration. It was also observed that in the membrane filtration of yeast cells with 20 wt% ethanol concentration, the cake structure became denser and the filtration resistance remarkably increased because of cellular destruction. Furthermore, the yeast cake exhibited a high compressibility in the solution containing a 20 wt% ethanol concentration. Therefore, the filtration rate of the alcoholic fermentation broth is not significantly improved by increased pressure due to the increase in the cake resistance.

## 1. Introduction

Microfiltration for the separation of particles has become increasingly important in various fields, including chemical and bio-industries. Membrane fouling is recognized as the most significant problem in the microfiltration process as it produces a drastic increase in the filtration resistance as the filtration progresses. Membrane fouling is caused by several factors such as the deposition of particles on the membrane surface referred to as the filter cake, and membrane pore-clogging and constriction [1,2,3,4,5]. Therefore, the mechanism underlying membrane fouling should be analyzed to control the microfiltration behavior of particles.

In the downstream processing of biotechnological products, microfiltration is used to remove the microbial cells from the fermentation broth. Because the impact of cake formation is one of the most significant factors influencing membrane fouling, the filtration behavior of the fermentation broth is strongly affected by the characteristics of the filter cake formed by the accumulation of cells on the membrane surface [6,7,8,9]. Therefore, knowledge of the cake structure provides valuable information for the design and operation of the filter equipment and membrane. During the alcohol fermentation process, the cake characteristics are considered to be complicated because microbial cells are strongly influenced by external factors such as filtration pressure and alcohol concentration. Yeast is the best-known ethanol-producing microorganism that is responsible both for the production of alcoholic beverages as well as bioethanol that is produced for use as a renewable fuel. In bioethanol production by yeast fermentation, attempts have been made to increase the ethanol concentration while reducing the required energy [10,11]. On the other hand, it has been reported that the dead yeast cells are observed in the fermentation broth containing a 20 vol% ethanol concentration [12]. Therefore, it is important to examine the filtration behaviors and cake characteristics of yeast under higher alcohol concentrations. Moreover, it is also necessary to obtain the dependences of the cake characteristics such as the average specific resistance and average porosity on the filtration pressure since yeast cell cakes have high compressibility [7,8,9].

Microfiltration is realized at the cross-flow mode or the dead-end mode with periodical backwashing to prevent the membrane from being fouled with deposits [13,14,15,16]. Redkar and Davis [17] observed that the initial transient flux decline in the cross-flow filtration of yeast follows the dead-end filtration theory. Furthermore, numerous investigations show that the resistances of yeast cakes both in cross-flow and dead-end filtration are similar [18,19]. As the most severe membrane fouling occurs in the dead-end mode, it is desirable to examine dead-end microfiltration behaviors in detail to clarify the role of the filter cake. Recently, several dead-end mode filtration tests have been proposed to accurately and simply determine the cake characteristics [8,20,21,22,23,24,25,26]. Among the cake characteristics, both the average specific cake resistance and average cake porosity, being related to the filterability, are recognized as the most important factors controlling membrane filtration behaviors. Murase et al. [20] developed a technique for accurately determining the average cake porosity in constant-pressure filtration based on the principle of a sudden reduction in the filtration area of the cake surface during the course of filtration. Iritani et al. [26] suggested a novel method for determining the average specific resistance and average porosity of filter cake, overlying the membrane in dead-end filtration, from only the flux decline behavior observed in two membrane filtration tests without using any specially designed filtration cells. It is considered that these filtration tests are also advantageous to the evaluation of the average specific resistance and average porosity of microbial cakes [9].

From the various approaches applied for dead-end microfiltration of microbial suspensions, both cells and metabolites have been shown to have significant effects on membrane fouling [6,7,8,9,27,28,29,30,31,32]. However, the cake characteristics and the effect of alcohol on yeast cake during membrane filtration of the alcoholic fermentation broth is not completely understood. In this study, we evaluated the membrane filtration properties, particularly the cake characteristics, of a yeast suspension containing ethanol in the dead-end mode, and investigated the effects of pressure and ethanol concentration on the separation of yeast. The results of this study, particularly regarding the effect of cell deformability on the cake characteristics in alcohol solutions, provide a promising strategy for efficient microfiltration that could be utilized for the production of bioethanol.

## 2. Materials and Methods

### 2.1. Materials

Sake Yeast NBRC2347 was used in this study. The yeast cells were inoculated into 200 mL of a medium (1% glucose, 0.5% peptone, 0.3% yeast extract, 0.3% malt extract) contained in a 500 mL Erlenmeyer flask. The flask was shaken at 200 rpm and 30 °C in a shaking incubator (BR-33FL, Taitec Corp., Saitama, Japan) to prepare the cell suspension culture. After cultivation, cell suspensions were centrifuged for 15 min at 3000 rpm, and the cells were washed twice by resuspending and centrifuging them in the Mcllvaine buffer (10 mM citric acid–20 mM Na_2_HPO_4_, pH 7.0) containing ethanol [33]. They were again suspended in the buffer and used for microfiltration experiments. The cell concentration *s*_c_ was determined by modifying the method of Ju and Ho [34] and expressed as the wet weight of cells per unit weight of the suspension.

### 2.2. Experimental Apparatus and Technique

**Figure 1:**
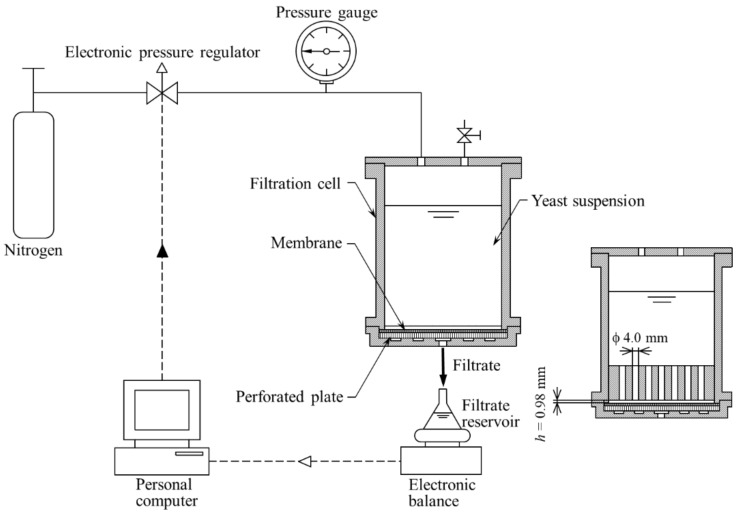
Schematic of the apparatus for microfiltration test.

Microfiltration experiments were conducted in a dead-end filtration mode using yeast suspensions containing ethanol at various concentrations (0, 5, 10, 15, and 20 wt%) under conditions of a constant pressure varying over the range from 1 to 500 kPa by adjusting the applied filtration pressure. Two types of unstirred batch filtration cells were utilized. A regular filtration cell was used to obtain a thick filter cake. To evaluate the average porosity *ε*_av_ of the filter cake, a filtration cell was used that was specifically designed for this study. In the case of this filtration cell, the filtration area was suddenly reduced from 16.88 to 2.58 cm^2^ by constructing an orifice structure at a distance *h* of 0.98 mm from the membrane surface, as shown in Figure 1. The filter medium used was a microfiltration membrane, made of a mixed cellulose ester, with a nominal pore size of 0.45 μm, supplied by Advantec Toyo Corp., Tokyo, Japan. The filtrate was collected in a reservoir placed on an electronic balance (Shimadzu Corp., Kyoto, Japan) connected to a personal computer to collect and record the mass versus time data. The weights were converted to volumes using density correlations, and the filtration rate *J*_v_ (=d*v*/d*θ*) at various volumes was computed by numerical differentiation of volume *v* versus time *θ* data. The filtrate was analyzed using a spectrophotometer (UV-1800, Shimadzu Corp.). The experiments were performed more than three times to ensure the reproducibility of the results. The cell cake was coated with gold using a sputter coater (MP-19020NCTR, JEOL Ltd., Tokyo, Japan), and an image of the bottom surface of the cake was obtained using a scanning electron microscope (JCM-5000, JEOL Ltd.).

## 3. Results and Discussion

### 3.1. Membrane Filtration Properties

**Figure 2:**
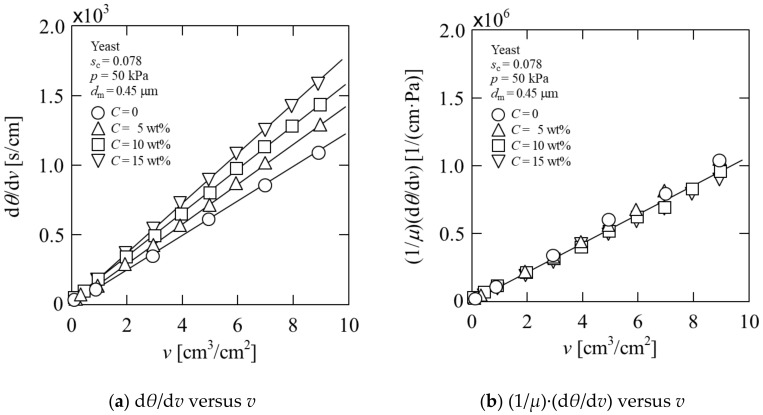
Microfiltration behavior of yeast suspension (*C* = 0–15 wt%): (**a**) d*θ*/d*v* versus *v*; (**b**) (1/*μ*)·(d*θ*/d*v*) versus *v*.

The cake filtration rate (d*v*/d*θ*) under constant pressure can be generally represented by the Ruth filtration rate equation [35]:

(1) $$\frac{d\theta }{dv}=\frac{2}{K_{v}}\left(v+v_{m}\right)=\frac{\mu \rho s_{c}\alpha_{\mathrm{av}}}{p\left(1-ms_{c}\right)}\left(v+v_{m}\right)$$

where *θ* is the filtration time, *v* is the cumulative filtrate volume per unit membrane area, *K*_v_ is the Ruth coefficient of constant pressure filtration, *v*_m_ is the fictitious filtrate volume per unit membrane area, equivalent to the flow resistance of membrane, *μ* is the viscosity of the filtrate, *ρ* is the density of the filtrate, *s*_c_ is the mass fraction of wet cells in suspension, and *p* is the applied filtration pressure. In Figure 2a, the filtration behavior of the yeast suspension is plotted as the reciprocal filtration rate (d*θ*/d*v*) against the cumulative filtrate volume *v* per unit membrane area. The plots are virtually linear in accordance with Equation (1) during filtration, and the *Y*-axis intercept near the origin indicates that the cake resistance is significantly higher than the membrane resistance. Consequently, the average specific cake resistance *α*_av_ that is a measure of the filterability of the suspension, can be calculated as:

(2) $$\alpha_{\mathrm{av}}=\frac{2}{K_{v}}\cdot \frac{p\left(1-ms_{c}\right)}{\mu \rho s_{c}}$$

The variable *m* indicates the average ratio of the mass of wet cake to the mass of dry cake and is related to the average porosity *ε*_av_ of the filter cake as:

(3) $$m=1+\frac{\rho \varepsilon_{\mathrm{av}}}{\rho_{c}\left(1-\varepsilon_{\mathrm{av}}\right)}$$

where *ρ*_c_ is the true density of the wet cells. It is assumed that the internal water of the cells is not drained during the filtration period [36].

In Figure 2a, the slope of the plot increases with increasing ethanol concentration *C* in the yeast suspension, indicating that the filtration resistance becomes higher from the existence of ethanol. As can be seen in Equation (1), the value of the slope is influenced by the viscosity of the filtrate, and it is well-known that the viscosity of the liquid changes when the ethanol content changes. Therefore, in Figure 2b, the constant pressure filtration data are replotted in the form of (1/*μ*)·(d*θ*/d*v*) against the cumulative filtrate volume *v* per unit membrane area, in accordance with Equation (4):

(4) $$\frac{1}{\mu }\cdot \frac{d\theta }{dv}=\frac{\rho s_{c}\alpha_{\mathrm{av}}}{p\left(1-ms_{c}\right)}\left(v+v_{m}\right)$$

Flux decline behaviors were put in a similar form for 0–15 wt% ethanol concentrations. This indicates that the cake resistance does not change with or without ethanol. The plot in Figure 2b is useful for representing the filtration behavior in the case where the liquid viscosity is variable.

**Figure 3:**
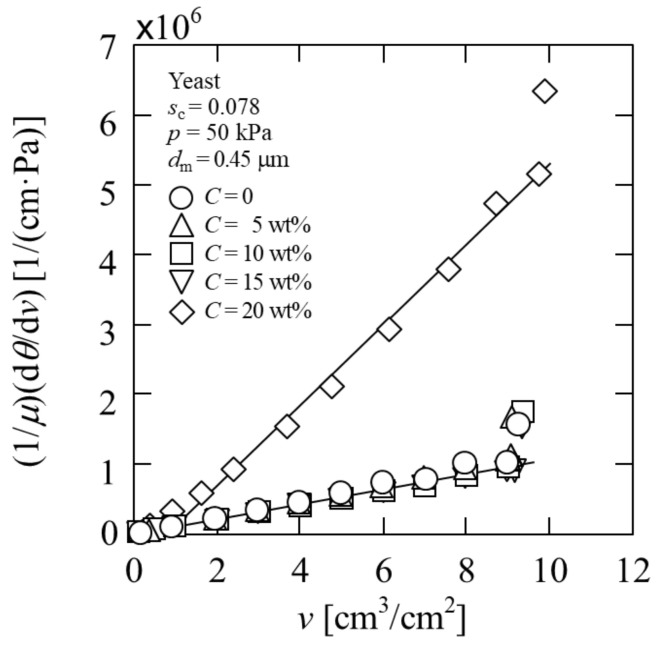
Microfiltration behavior of yeast suspension (*C* = 0–20 wt%).

In contrast, a severe flux decline is observed for the suspension with 20 wt% ethanol concentration, as shown in Figure 3. In this case, the increase in the slope of the plot is attributed to the characteristics of the cake formed on the membrane surface, and indicates that the values of *α*_av_ and *m* have changed significantly, as can be inferred from Equation (4). This flux decline behavior is confirmed even at the ethanol concentration of 25 wt% (data not shown), and is considered to be a characteristic behavior when the alcohol concentration is high. It is known that the increased concentration of ethanol influences the cell membrane fluidity and is toxic to the membrane proteins, leading to cell growth inhibition, and even death [37]. That is, the effect of high-concentration alcohol on cells has evidently induced changes in the cake characteristics. Although there have been attempts to increase the ethanol concentration in bioethanol production [10,11], the microfiltration test results indicate that the filterability is significantly reduced under high ethanol concentrations.

### 3.2. Characteristics of Yeast Cell Cake

The average specific resistance *α*_av_ and average porosity *ε*_av_ of the yeast cell cake were measured based on cake filtration theory. To evaluate the average porosity *ε*_av_ of the filter cake, a specifically designed filtration cell was used. Once the surface of the filter cake reaches the position where the inner cross-sectional area of the filter chamber is suddenly reduced from 16.88 to 2.58 cm^2^, the filtrate flow rate decreases dramatically because of the decrease in the surface area of the filter cake. Consequently, the plot of d*θ*/d*v* against *v* calculated by filtration area 16.88 cm^2^ shows a significant deviation from the previous linear relationship, as shown in Figure 3. The thickness *L* of the filter cake at the transition point is equal to the distance *h* from the membrane surface to the orifice structure [20]. With respect to the overall mass balance in filtration, the distance *h* is directly related to the filtrate volume *v*_t_ per unit membrane area at the transition point based on the following equation [9]:

(5) $$w_{t}=\frac{\rho s_{c}}{1-ms_{c}}v_{t}=\rho_{c}\left(1-\varepsilon_{\mathrm{av}}\right)h$$

where *w*_t_ is the mass of wet cells per unit membrane area at the transition point. Therefore, substituting Equation (3) into Equation (5) results in the following equation [9]:

(6) $$\varepsilon_{\mathrm{av}}=\frac{\rho_{c}\left(1-s_{c}\right)h-\rho s_{c}v_{t}}{\rho_{c}\left(1-s_{c}\right)h+\rho s_{c}h}$$

**Figure 4:**
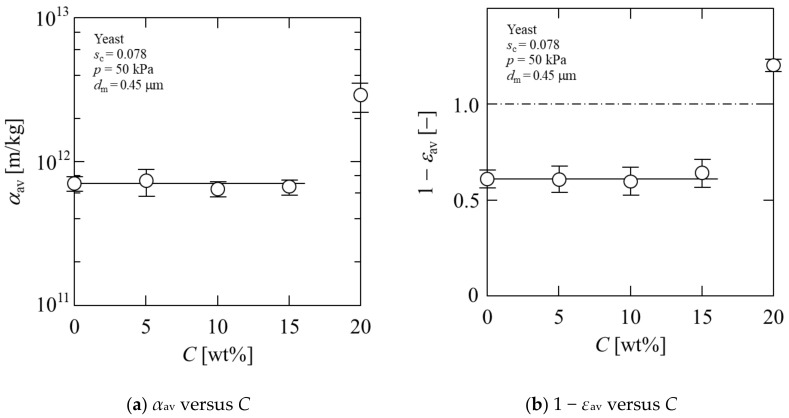
Relationship between cake characteristics and ethanol concentration: (**a**) *α*_av_ versus *C*; (**b**) 1 − *ε*_av_ versus *C*.

Figure 4 represents the relationship between the cake characteristics and ethanol concentration *C*. At an ethanol concentration of 15 wt%, the value of the average specific cake resistance *α*_av_ almost remained unchanged as the concentration increased, as shown in Figure 4a. However, *α*_av_ at 20 wt% was much higher than that at 0–15 wt%, and the value was changed by one order of magnitude. In Figure 4b, the solidosity (1 − *ε*_av_) is plotted against the ethanol concentration. The result was similar to that of *α*_av_, and the value of solidosity at *C* = 20 wt% was different from those at the other ethanol concentrations. In this study, we calculated the average cake porosity *ε*_av_ using Equation (6). The gap between cells was considered as the void through which water drains. Therefore, when the solidosity (1 − *ε*_av_) exceeded 1.0, the internal water of the yeast cell was drained. The value of *ε*_av_ at 0–15 wt% was 0.35–0.4, which was in agreement with the results of other studies [14,33]. In contrast, *ε*_av_ at 20 wt% was extremely low, indicating a significant increase in the filtration resistance.

**Figure 5:**
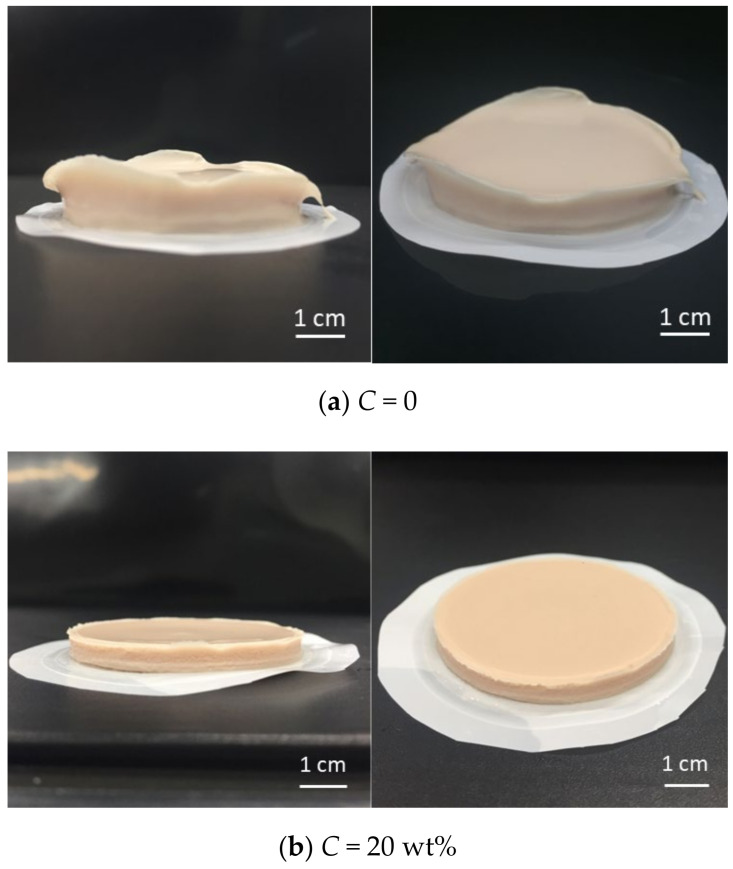
Photo of filter cake (*s*_c_ = 0.078, *p* = 50 kPa): (**a**) *C* = 0; (**b**) *C* = 20 wt%.

Figure 5 shows photographs of the yeast cake when the ethanol concentration *C* is 0 wt% and 20 wt%. The thickness of the yeast cake at *C* = 20 wt% (Figure 5b) was remarkably different from that at *C* = 0 (Figure 5a), and the dehydration progressed and a hard cake was formed. The texture of the cake is clearly different, and it is speculated that the state of the yeast cells that comprise the cake has also changed. This result is consistent with what is inferred from the solidosity data in Figure 4b.

**Figure 6:**
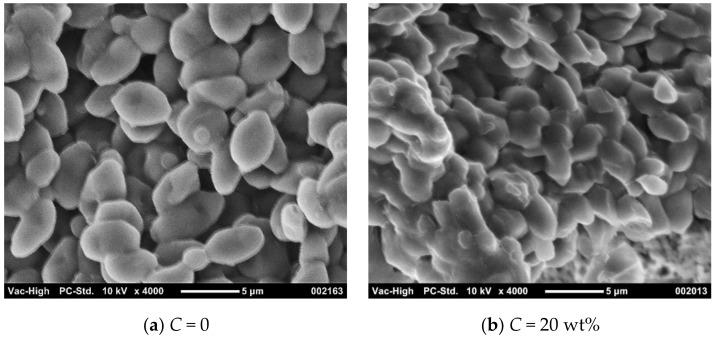
Electron micrograph of filter cake (*s*_c_ = 0.078, *p* = 50 kPa): (**a**) *C* = 0; (**b**) *C* = 20 wt%.

Figure 6 shows electron micrographs of the bottom surface of the cake. Looking at the yeast cells that comprise the cake, each cell can be confirmed at *C* = 0, whereas the cells are bound to each other at *C* = 20 wt%. It is known that ethanol is destroys the membrane structure of yeast cells [37], and Urbanczyk et al. [12] observed that the percentage of dead cells increased with an increasing ethanol concentration of the fermentation broth. It can be seen that the high concentration of ethanol has damaged the cell membrane and has affected the cake structure.

**Figure 7:**
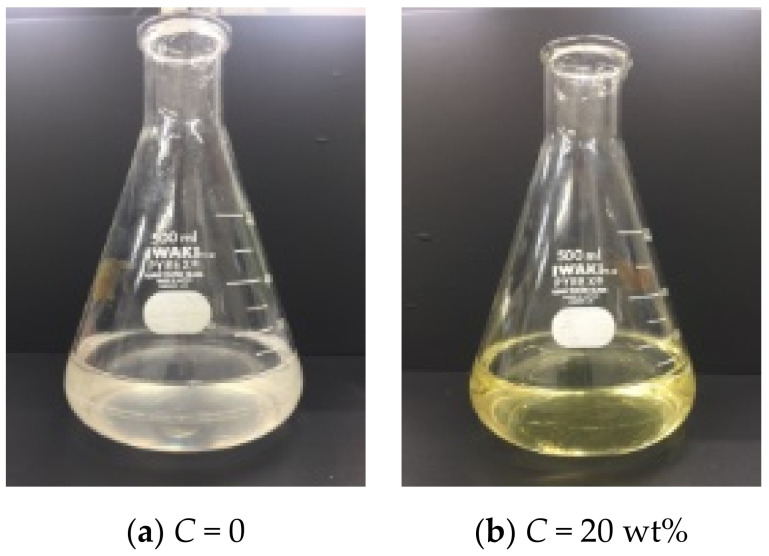
Photo of filtrate (*s*_c_ = 0.078, *p* = 50 kPa): (**a**) *C* = 0; (**b**) *C* = 20 wt%.

**Figure 8:**
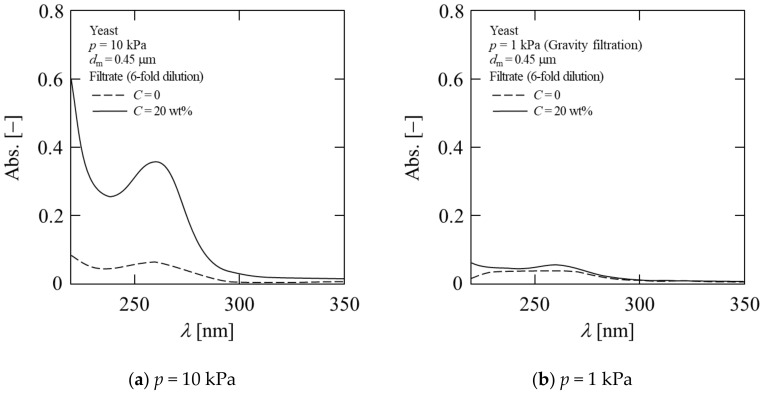
Spectrum of filtrate: (**a**) *p* = 10 kPa; (**b**) *p* = 1 kPa.

Furthermore, a clear difference was observed in the filtrate at *C* = 20 wt%. As can be seen from the photograph shown in Figure 7, yellow coloring is observed in the filtrate at *C* = 20 wt%, although it is not observed at *C* = 0. It is possible that metabolites have been discharged from the yeast cells. The filtrate was analyzed with a spectrophotometer; the spectrum is shown in Figure 8. At a filtration pressure of 1 kPa, there was no difference based on the ethanol concentration; however, at a filtration pressure of 10 kPa, a peak was observed near 260 nm at *C* = 20 wt%. This suggests that the cells were destroyed, and intracellular metabolites such as nucleic acid were discharged into the filtrate under a pressure above 10 kPa at *C* = 20 wt%. This phenomenon has a great influence on the quality of alcoholic beverages. Furthermore, with respect to the dehydration of microbial cakes, it is considered that such findings may be applied to the establishment of an efficient dehydration method for microbial cells. From these results, it is confirmed that the cake structure becomes denser and the filtration resistance is remarkably increased during the filtration of yeast suspensions with high alcohol concentrations.

**Figure 9:**
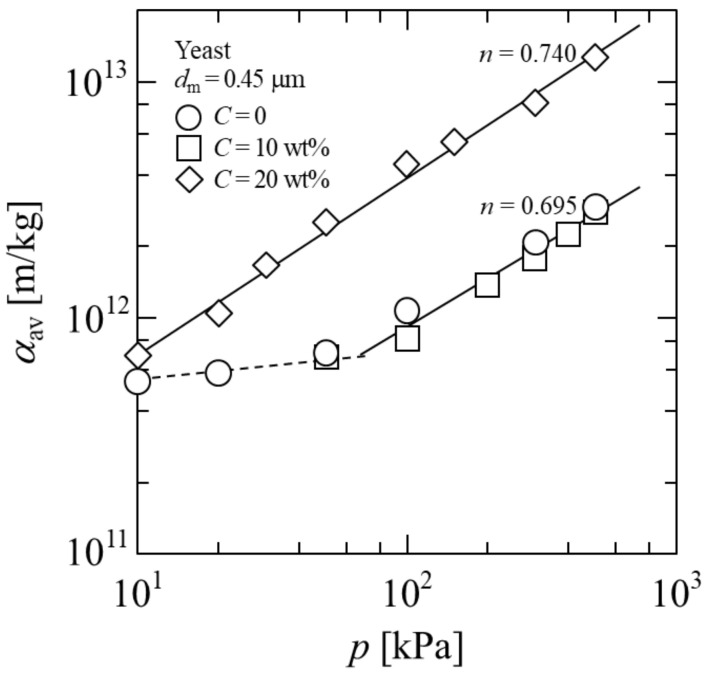
Logarithmic plot of average specific cake resistance against applied pressure.

In Figure 9, the average specific filtration resistance *α*_av_ of the yeast cake is logarithmically plotted against the pressure *p*. In general, it is possible to empirically represent *α*_av_ by the power function of *p* for compressible filter cakes as follows [38]:

(7) $$\alpha_{\mathrm{av}}=\alpha_{1}p^{n}$$

where *α*_1_ and *n* are the empirical constants, and *n* is a measure of the compressibility of the filter cake. However, in the case of microbial cake, it is known that Equation (7) can be applied above a certain pressure [6,31], and such behavior is observed at C = 0 and 10 wt%. In contrast, at C = 20 wt%, a particular behavior is observed that allowed Equation (7) to be applied in the pressure range from 10 to 500 kPa. It was clarified that in the high-concentration alcohol solution, the yeast cake was compressed even under low pressure, as in the case of high pressure. The values of the compressibility coefficient *n* were calculated to be 0.740 and 0.695 for the yeast cake at C = 20 wt% and C = 0, respectively. It should be noted that the filter cake formed at C = 20 wt% shows a higher compressibility than that formed at C = 0. That is, the yeast cell present in the high-concentration alcohol solution causes a high compressibility of the filter cake. As a result, the filtration rate of the alcoholic fermentation broth is not significantly improved by the increased pressure due to the increase in the cake resistance. These findings will be useful for determining an efficient filtration pressure.

## 4. Conclusions

The microfiltration properties of yeast suspensions containing ethanol at various concentrations were evaluated based on the compressible cake filtration theory. The cake structure became denser and the filtration resistance was increased remarkably for high ethanol concentrations of the suspension. The high-concentration ethanol influenced the yeast cell membrane, and the destroyed and deformed cells induced changes in the cake characteristics. The discharge of intracellular components into the filtrate has a great influence on the quality of alcoholic beverages. It was also clarified that in the high-concentration ethanol solution, the yeast cake was evidently compressed even under low pressure, and the filtration rate is not so much improved by the increase in the applied pressure. The results of the effect of cell deformability on cake characteristics in alcohol solution provide a promising strategy for an efficient microfiltration that could be employed for the production of bioethanol.

## Data Availability

Not applicable.
